# Patients’ view on gene therapy development for lysosomal storage disorders: a qualitative study

**DOI:** 10.1186/s13023-022-02543-y

**Published:** 2022-10-21

**Authors:** Eline C. B. Eskes, Cathrien R. L. Beishuizen, Eleonore M. Corazolla, Tessa van Middelaar, Marion M. M. G. Brands, Hanka Dekker, Erica van de Mheen, Mirjam Langeveld, Carla E. M. Hollak, Barbara Sjouke

**Affiliations:** 1 Amsterdam UMC, University of Amsterdam, Endocrinology and Metabolism, Meibergdreef 9, Amsterdam, The Netherlands; 2 Amsterdam Gastroenterology Endocrinology Metabolism, Inborn Errors of Metabolism, Meibergdreef 9, Amsterdam, The Netherlands; 3 Amsterdam UMC, University of Amsterdam, General Practice, Meibergdreef 9, Amsterdam, The Netherlands; 4 Amsterdam UMC, University of Amsterdam, Laboratory Genetic Metabolic Diseases, Meibergdreef 9, Amsterdam, The Netherlands; 5 Amsterdam UMC, University of Amsterdam, Neurology, Meibergdreef 9, Amsterdam, The Netherlands; 6 Amsterdam UMC, University of Amsterdam, Emma Children’s Hospital, Department of Pediatrics, Division of Metabolic Diseases, Meibergdreef 9, Amsterdam, The Netherlands; 7 VKS, The Dutch Patient Association for Inherited Metabolic Diseases, Zwolle, The Netherlands; 8 Fabry Support and Information Group the Netherlands (FSIGN), Drachten, The Netherlands

**Keywords:** Lysosomal storage diseases, Gaucher disease type 1, Fabry disease, Mucopolysaccharidosis type III, Gene therapy, Qualitative research, Focus group discussions

## Abstract

**Introduction:**

Several new treatment modalities are being developed for lysosomal storage disorders (LSDs), including gene therapy. As the currently available treatment options and their influence on disease progression differ greatly within the spectrum of LSDs, willingness to undergo gene therapy might vary among patients with LSDs and/or their representatives. The width of the LSD spectrum is illustrated by the differences between type 1 Gaucher disease, Fabry disease and Mucopolysaccharidosis type III (MPS III). For type 1 Gaucher and Fabry disease several therapies are available, resulting in a near normal or improved, but individually varying, prognosis. No treatment options are available for MPS III.

**Aim:**

To identify factors influencing patients’ and/or their representatives’ decisions regarding undergoing gene therapy.

**Methods:**

Focus group discussions and semi-structured interviews were conducted with patients with type 1 Gaucher disease, Fabry disease and MPS III. Parents of MPS III patients were included as patients’ representatives.

**Results:**

Nine Gaucher patients, 23 Fabry patients, two adult MPS III patients and five parents of MPS III patients participated in the study. The five main themes that arose were: *outcome of gene therapy, risks and side effects, burden of gene therapy treatment, current situation* and *ethical aspects*. Participants’ views ranged from hesitance to eagerness to undergo gene therapy, which seemed to be mostly related to disease severity and currently available treatment options. Severe disease, limited treatment options and limited effectiveness of current treatment augmented the willingness to choose gene therapy. Gaucher and Fabry patients deemed the *burden of treatment* important. Fabry and MPS III patients and parents considered *outcome* important, suggesting hope for improvement. When asked to rank the factors discussed in the focus group discussions, Gaucher patients ranked *outcome* low, which could indicate a more cautious attitude towards gene therapy.

**Conclusion:**

This study underlines the importance of exploring patients’ needs and expectations before using limited resources in the development of therapies for patient groups of which a significant subset may not be willing to undergo that specific therapy.

**Supplementary Information:**

The online version contains supplementary material available at 10.1186/s13023-022-02543-y.

## Introduction

The group of lysosomal storage disorders (LSDs) includes more than 60 genetic diseases, caused by a deficiency of a specific enzyme, transporter or cofactor [1]. This results in accumulation of macromolecules that cannot be properly degraded, causing chronic, progressive, multi-system disorders with a broad clinical spectrum [2]. Disease severity, rate of progression and the availability of effective treatment vary greatly between the different disorders [2, 3].

Several therapies are available for some of the LSDs, for instance enzyme replacement therapy (ERT), substrate reduction therapy (SRT) and chaperone therapy [3, 4]. Their variable success in modulating visceral disease manifestations might in part be explained by the amount of irreversible tissue damage at the start of therapy. Moreover, the therapies that are currently available are generally not able to cross the blood–brain barrier and as such have no ability to alter neurological manifestations [3, 5, 6]. Therefore there is an unmet need regarding therapeutic options for LSDs with neurological manifestations and/or irreversible manifestations in early stages of disease.

The width of the spectrum of LSDs regarding phenotype and therapeutic options is illustrated by the differences between type 1 Gaucher disease (OMIM #230800), Fabry disease (OMIM #301500) and Mucopolysaccharidosis type III (MPS III, OMIM #252900). For two of them therapy is available: ERT and SRT for type 1 Gaucher disease and ERT and chaperone therapy for Fabry disease [7, 8]. When treated with ERT, type 1 Gaucher patients have a near normal life expectancy with minimal symptoms of the condition [4]. In some Fabry patients, long-term treatment with ERT seems effective in reducing disease progression, especially renal manifestations [9]. However, a subset of patients still develop complications despite treatment [9]. Several therapeutic options have been studied for MPS III in clinical trials (e.g. ERT and SRT), but none of them resulted in an approved therapeutic option [10–13].

In the past, hematopoietic stem cell transplantation (HSCT) has been performed in patients with different LSDs. Because of its burdensome treatment procedure and variable success, it became superseded by other treatment modalities [14, 15]. Building on HSCT, the concept of gene therapy is to ameliorate the metabolic deficiency in target cells by introducing correct genetic material for the deficient enzyme. Since most LSDs are monogenetic disorders, this is a potential therapeutic strategy (see Box 1).

**Box 1:** Gene therapy treatment

Gene therapy has been researched for decades and is assumed to be a one-time treatment in which genetic material coding for the deficient protein is inserted in the nucleus of targeted cells, giving those cells the ability to produce that protein [16, 19]. Several approaches for delivery of the genetic material are applied which can be broadly categorized into in vivo and ex vivo [20, 21]
In the in vivo approach, specific tissues are targeted by using a virus as a vector [20, 21]. This is a relatively simple procedure. However, it might be that the virus used as a vector can only be used once in an individual because the immune system will recognize it a second time
The ex vivo approach is more elaborate and requires hematopoietic stem cells to be recruited from the patient and to be transduced with the correct DNA in vitro. The patient receives chemotherapy to clear residual hematopoietic stem cells, after which the transduced stem cells are reintroduced [20, 21]
Effectiveness depends on the ability of the transduced cells to release the enzyme into the circulation and whether the enzyme is then able to reach affected tissues. Long term effects of both approaches have not been extensively researched. Development of neutralizing antibodies and/or immune reactions might influence the long term treatment effects [22]

The first studies into gene therapy in humans were initiated in the 1990s, resulting in eleven gene therapy medicinal products currently approved by the European Medicine Agency, of which one is for a LSD [16, 17]. Currently, twenty nine gene therapy trials in LSDs including Gaucher type 1, Fabry disease and MPS III are registered as active, illustrating the rapid expansion of this area of research (18). As the available treatment options and their influence on disease progression differ greatly within the spectrum of LSDs, willingness to participate in a gene therapy trial might differ among LSD patients. The burden of existing therapies might influence this decision as well (ERT is administered intravenously, generally every two weeks, SRT and chaperone therapy are oral therapies). Since developing new therapies for rare diseases is a challenge because of the limited amount of patients that can potentially participate in trials, it is of importance to tailor the design of clinical trials to the needs of patients. This qualitative study aims to explore the needs and expectations of patients (or their parents) with type 1 Gaucher disease, Fabry disease or MPS III regarding the development of gene therapy. Insights from this study might guide clinicians, regulators and pharmaceutical companies in developing and prescribing therapies that best meet patients’ needs and expectations.

## Methods

### Expert panel

An expert panel was composed to discuss the design and methodology of the study. The expert panel consisted of three clinical experts specialized in metabolic diseases in adults (ML, CH and BS), a clinical expert specialized in inherited metabolic diseases in children (MB) and patient representatives (HD, EM). This panel was involved throughout the study as described below.

### Participants and settings

The expert panel identified three LSDs representing the width of the spectrum in terms of disease severity and therapeutic options: Gaucher disease type 1, Fabry disease and MPS III. The Amsterdam UMC, location AMC is the national referral center for all three LSDs, so almost all patients in the Netherlands are monitored at our outpatient clinic. All adult patients with type 1 Gaucher or Fabry disease able to participate in group discussions and/or interviews were invited to take part in the study. Since the included children with MPS III were not able to participate because of either their young age and/or cognitive impairment due to their disease, their parents were invited as their representatives. Exclusion criteria were a recent diagnosis (diagnosed with the respective LSD < 1 year before the study), insufficient Dutch language proficiency or legal incompetence. All eligible patients (or their parents in case of MPS III children) who are monitored at our outpatient clinic were approached with a letter inviting them to participate in this study. Besides the information letter, they received concise background information on gene therapy research development for their disease and on the two different delivery approaches of gene therapy. All participants provided written informed consent.

Due to the COVID-19 pandemic, the group discussions and interviews were conducted online using Microsoft Teams (version 1.4). Group size was set to a maximum of five participants per session. The Consolidated criteria for reporting qualitative research (COREQ) reporting guidelines were adhered to in the reporting of this study [23].

### Data collection

In November 2020, ten online focus group discussions took place. The groups were divided by disease to allow disease-specific discussions to arise between participants. In case of Fabry disease the focus groups were divided by disease phenotype, based on sex and type of mutation. The focus group discussions were moderated by CB, who is a MD PhD experienced in qualitative research and moderation of focus groups. Assistant moderators were TM, a MD PhD experienced in qualitative research, and EE, a MD PhD-student researching LSDs. CB and TM were not involved in clinical care of the participants, nor was any of the moderators directly involved in the development of or in clinical studies on gene therapy, in order to ensure an unbiased and open discussion. The moderators used a flexible topic list (see Additional file 1) to guide the discussion, asking open-ended questions. Discussed topics were: associations with gene therapy, needs and preferences for future gene therapy and factors regarded important in deciding to undergo gene therapy. BS, a MD PhD involved in the clinical care of all three diseases, attended all focus groups without active participation. Halfway through each session, BS answered questions about the background information on gene therapy to ensure that all participants’ questions were answered. In the MPS III focus groups, this was done by MB and BS. At the end of the focus group discussion, factors influencing the decision to undergo gene therapy were summarized and participants were asked to rank these by importance.

After the first focus group discussion, refinement of the topic list took place to ensure an in-depth exploration of all topics encountered. At the end of all focus groups, data saturation had been reached for Gaucher and Fabry disease, since no new themes emerged in the last discussions. For MPS III, this was not yet the case. Therefore, we conducted three semi-structured interviews with one parent of an MPS III patient and two adult MPS III patients, which resulted in saturation. Additionally, three Fabry patients were interviewed who could not participate in the focus groups for practical reasons as well as one Gaucher patient who had already participated in the group discussion but for whom the investigators had additional questions. The MPS III interviews were conducted by CB with support from BS, the Gaucher and Fabry interviews were conducted by EE with support from EC. For the interviews, the topic list was adjusted and shortened in order to explore the most important factors based on the focus group discussions in more depth (see Additional file 2). Similar to the focus group discussions, at the end of each interview factors influencing the decision whether to choose for gene therapy as mentioned by the participant were summarized and the participant was asked to rank them. All focus groups and individual interviews were audio-recorded, transcribed verbatim and field notes were taken.

### Coding and analysis

**Fig. 1:**
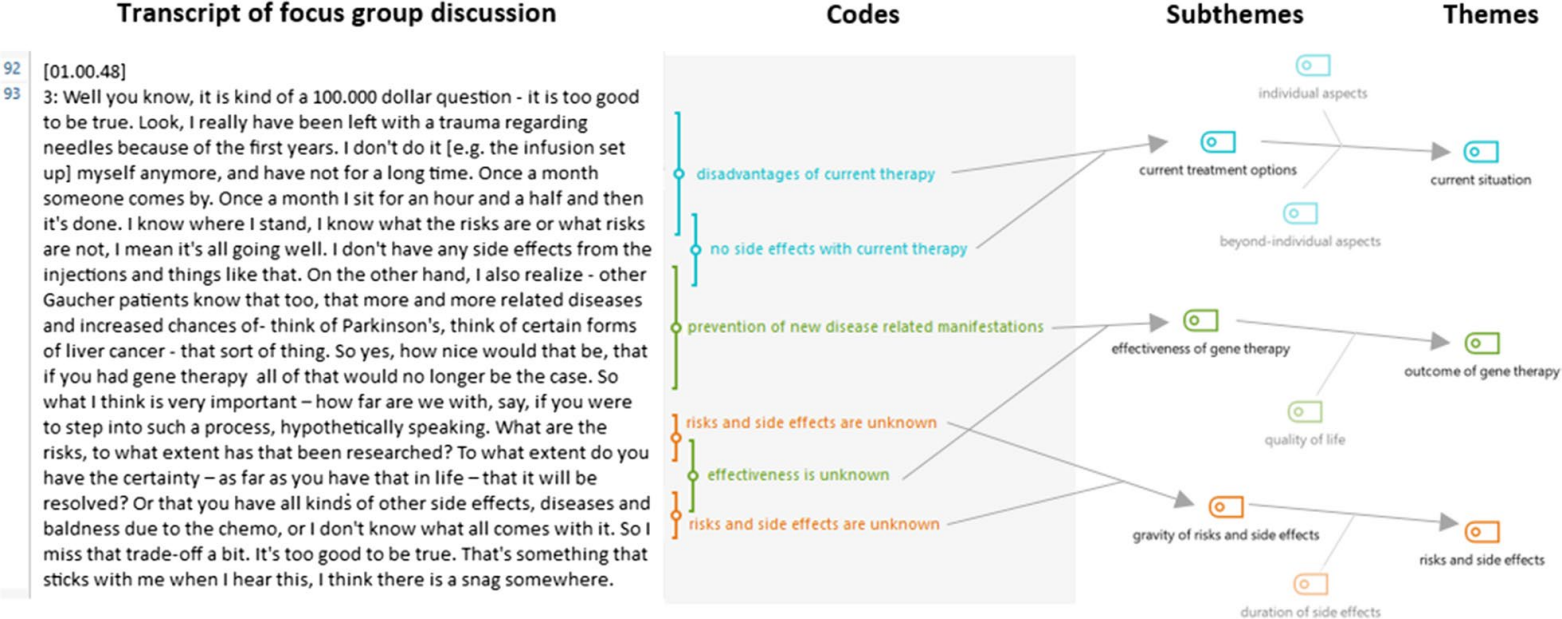
**Coding in MAXQDA**. On the left the transcript of one of the focus groups discussions and the codes that were derived from the text. On the right the coding tree with subthemes that are grouped into themes

Three researchers (EE, CB and EC) thematically analyzed the data in an iterative process, using MAXQDA software (version 20.3.0, www.maxqda.com). First, the transcripts were independently coded by EE and EC using an inductive approach. Next, EE and EC reviewed each other’s codes to achieve inter-observer agreement. Hereafter the codes were discussed with CB resulting in a uniform coding scheme for all transcripts. The codes were structured into a coding tree of main themes and subthemes (see Fig. 1 for an illustration of this process). Themes were derived from the data and were not assumed a priori. Finally, the results were discussed with BS and conclusions were checked in the transcripts. Summaries per disease were made and emailed to the participants to verify the conclusions, not resulting in significant changes.

### Ethical approval and privacy

The study protocol was reviewed by the Medical Ethics Committee of the Amsterdam UMC location Academic Medical Center and ethical approval of this study was granted. A data protection impact assessment was performed under supervision of the privacy officer of the Amsterdam University Medical Centers to ensure compliance with the data protection obligations under the General Data Protection Regulation. All participants provided written informed consent.

## Results

### Participants, focus groups and interviews

Thirty nine patients participated in a focus group or an interview: nine Gaucher patients, 23 Fabry patients, two MPS III patients with an attenuated phenotype (interviews) and five parents of (young) MPS III patients with or potentially developing severe manifestations, from five different families. Participation rates for the three groups were respectively 15, 14 and 21%. This resulted in two focus groups with Gaucher patients, six with Fabry patients (one with classical male patients, four with non-classical male and/or classical female patients and one with non-classical female patients) and two with parents of MPS III patients. All patients invited to participate in an interview were willing to, resulting in interviews with one Gaucher patient, three Fabry patients, two MPS III patients and one parent of an MPS III patient. The duration of focus group discussions varied between 72 and 119 min, interviews varied between 19 and 73 min.

Demographics, treatment and disease subtypes of the patients who participated in the focus group discussions and interviews are summarized in Table 1. All Gaucher and Fabry patients lived independently. The MPS III children were all dependent of their parents or other caregivers. Ninety five percent of the participants was of Western European descent. All therapies that were used by the participants of this study are reimbursed by the government, with the exception of the therapies within the context of a clinical trial (n = 3).

**Table 1:** Characteristics of the participants

	Gaucher	Fabry	MPS III	
			Patients	Parents^#^
*Demographics*				
Number	9	23	2	5
Female sex (n)	3	17	0	3
Age (median, range)	59 (49–72)	50 (20–64)	20,23	10 (18m–26y)
*Current treatment*				
ERT (n)	9	17	0	0
SRT (n)	0	1	0	0
Investigational treatment (Clinical trial) (n)	0	1	0	2
No disease specific treatment (n)	0	4	2	3
*Disease subtype*				
Gaucher type 1 (n)	9	–	–	–
Classical Fabry disease (n)	–	19	–	–
Non-classical Fabry disease (n)	–	4	–	–
MPS IIIA (n)	–	–	2	4
MPS IIIC (n)	–	–	0	1

*ERT* enzyme replacement therapy, *SRT* substrate reduction therapy

^#^For parents of MPS children the characteristics of their child are displayed

### Views on gene therapy development

Patients from all three disease groups wanted to stimulate the development of gene therapy. They hoped that gene therapy would become a definite treatment option in the future, possibly even providing a cure for the diseases. However, when discussed whether they would choose gene therapy themselves, distinct differences between the patient groups as well as individual differences emerged. The five themes deemed most important in the decision making process were: *outcome of gene therapy*, *risks and side effects*, *burden of gene therapy treatment*, *current situation* and *ethical aspects* (see Table 2). These themes were attributed different weight by each patient group and are described below.

**Table 2:** Themes and subthemes of the factors contributing to the choice to undergo gene therapy and the overall attitude/opinion of the patient group

Theme	Subtheme	Gaucher	Fabry	MPS III
Outcome of gene therapy	Effectiveness of gene therapy	Gene therapy should be as effective as current therapy	Hope for gene therapy to be more effective than current therapy	Every potential effect gives hope
	Quality of life			
Risks and side effects	Gravity of risks and side effects	Critical attitude	Acceptance	High acceptance
	Duration of side effects			
Burden of gene therapy treatment	Method of gene therapy	Critical attitude	Acceptance	High acceptance
	Practical aspects			
Current situation	Current treatment options	Good	Uncertainty	None
	Beyond-individual aspects	Important	Very important	Extremely important
	Individual aspects	Differ between group and have different impact		
Ethical aspects	Personal and societal aspects	Not disease specific		

### Outcome of gene therapy

The theme *outcome of gene therapy* can be divided into the subthemes *effectiveness* (i.e. prevention of clinical deterioration and improvement of life expectancy) and *quality of life*. In general, patients expected gene therapy to stabilize the disease, prevent progression and increase quality of life. Moreover, the hope was expressed that the disease would be cured by gene therapy; ideally it would be a one-time treatment resulting in lifelong endogenous enzyme production. Patients expected that especially young patients and patients diagnosed in the future will benefit from gene therapy, as they might have the possibility to undergo this therapy before having suffered irreversible organ damage.

“The fact that you could offer gene therapy to a new generation Gaucher patients […]. That is the goal of gene therapy for me. And for us [older Gaucher patients] it would just be nice to be able to do without the infusions every other week.” (participant 2, focus group (FG) Gaucher 2)

Gaucher patients did not expect much improvement regarding effectiveness compared to their current therapy, which is already quite effective. A factor that would improve quality of life would be to not depend on infusions every other week.

“I think for our disease it plays a role that we have an effective therapy. […] I do not expect much improvement in terms of health outcomes […]. Not having to get an infusion every two weeks, that would be the biggest advantage for me.” (participant 1, FG Gaucher 1)

Fabry patients hoped gene therapy will be more effective than current therapy options in preventing organ damage and complications. Additionally, they hope it will be more effective for complaints that ERT cannot solve such as pain and fatigue, which greatly influence their quality of life.

“My biggest wish is pain reduction, because enzyme replacement therapy currently does not achieve that for me. Pain reduction would be an ideal outcome.” (participant 4, FG Fabry 1)

For MPS III patients and parents, gene therapy was considered the only chance for a successful treatment. They hoped for stabilization of disease as well as improvement of life expectancy.

“You just go for it fully. They will only administer it [gene therapy] to people when it is relatively well tolerated, but there are still risks. […] I think it’s a difficult dilemma but I would choose to do it. It feels like a lifeline and hope for the future.” (participant 1, FG MPS III 1)

Parents of young MPS III patients mentioned that the psychological burden on the family would be relieved by stabilization of disease, as there would be less uncertainty about progression. In contrast, a parent of an older severely affected child with MPS III feared he would not be able to take care of his child when his life expectancy would increase:

“Life expectancy plays a role. If [my child with MPS III] got medication now and stayed stable in the current condition for 30 years, I would be 30 years older - who is going to take care of my child then? […] Normally you outlive your child [as a parent of a severe MPS III patient] but this way you have an additional thing to worry about. That is an important factor for me. I don’t want to put the burden on others. It is my responsibility to take care of my child the best I can, until the end.” (participant 1, FG MPS III 1)

### Risks and side effects

This theme can be divided into *severity* and *duration of risks and side effects*. In general, the risks and side effects of the ex vivo approach of gene therapy (e.g. nausea, temporarily impaired function of the immune system, bleeding) were considered more serious than those of the in vivo approach (e.g. possibility of long term use of immunosuppressive medication). Gaucher patients were the most critical, they would not accept a large margin of uncertainty regarding effectiveness, risks and side effects.

“If you have had [gene therapy] and [additional medication] is necessary, then you have no choice. But I don’t know if I would do [gene therapy] if I knew I had to take additional medication.” (participant 2, FG Gaucher 2)

Fabry patients expressed mixed feelings regarding risks and side effects, which seemed related to their disease burden combined with the course of disease in (older) family members.

“I have barely any complaints due to my illness. I can do my job and take care of my family, and feel energetic. The same as before I knew [I had Fabry disease]. […] Since the infusion therapy is going well and is easy to fit into my life, I prefer to wait for a little while longer.” (participant 1, FG Fabry 6)

“I have my father as an example of how ill you can get because of Fabry disease. That’s my perspective and that makes [gene therapy] feel less burdensome for me. It will definitely be hard, and you should not underestimate side effects, they will be unpleasant. But when I think of what might happen to me because of Fabry disease, I am positive towards the idea [of gene therapy].” (participant 4, FG Fabry 4)

Parents of MPS III patients were willing to accept a higher level of uncertainty regarding risks and side effect than patients with Fabry or Gaucher disease, since there is no alternative for their children. This is illustrated by a parent of a child that has already participated in an international trial for gene therapy:

“[My child with MPS III] is not ill. [In the trial we are currently partaking in] my child has to take medication which can cause all kind of side-effects. […] I have thought a lot about whether we should do it, since we did not know how bad the side-effects will be. […] If we had not done anything, we knew what would happen, so we realized we had to [participate in the trial]” (participant 2, FG MPS III 2)

Adult MPS III patients with attenuated disease were more hesitant, but were willing to accept mild or temporary side effects when there is a reasonable chance for improvement.

### Burden of gene therapy treatment

The theme *burden of treatment* comprises the *method of gene therapy* and *practical aspects*. In general, the ex vivo approach was estimated to be more burdensome than the in vivo approach, mainly because the ex vivo approach requires chemotherapy. Patients would weigh the burden of treatment against their health and physical condition at the moment they have to decide whether to undergo gene therapy.

Gaucher patients were critical about the chemotherapy required for the ex vivo approach and found that too invasive. For them age, physical condition and the time it would take to recover from the procedure were of importance in the decision, as was the case for elderly Fabry patients.

“[I would take into account] the current state of my health. I value the time with my children very much. And if it became clear that I suffer from heart failure for example, […] I would consider a year (i.e. hypothetic period the patient might be suffering from side effects) too long.” (interview Fabry 1)

Younger Fabry patients would accept frequent visits to the hospital but expressed concerns about practical aspects regarding work and family life if they would be incapacitated for more than a few weeks.

Parents of MPS III patients were willing to accept the burden of treatment, which is even higher in MPS III than in Gaucher and Fabry in the current trials because of the need of intracerebral administration. They were willing to let their child undergo brain surgery, travel abroad for the procedures and enter a long trajectory with repetitive brain scans, blood draws and other assessments. One parent had already entered such a trajectory with an MPS III child in the context of an ongoing international clinical trial and shared that it was very burdensome but considered it worth it.

“A hole was drilled in [my child’s] skull and the gene was administered in the cerebral ventricles. […] So much is done to your child. […] It is hard to witness, but if it has advantages you hope that later you will be able to explain that it was necessary.” (participant 1, FG MPS III 2)

The parent of an older child, who already had irreversible manifestations of MPS III, was more reluctant and reckoned the procedures might be too burdensome for the child. Adult MPS III patients considered the treatment worth it if stabilization of disease would be achieved.

### Current situation

This theme is broad and can roughly be divided into the subthemes *current treatment options*, *individual aspects* (e.g. health perception and current disease burden) and *beyond-individual aspects* (e.g. societal aspects and experiences with the disease in family members). The most important subtheme was *current treatment options* and their burden as experienced by the patient. Besides individual factors, the idea to contribute to a better treatment for young patients or future generations was a strong motivation. Gaucher patients were most reluctant, since most of them have participated in clinical trials in the past and they found it is the younger generation’s turn now.

“Young patients with Gaucher should realize that they suffer from a disease that could have severe manifestations in the future and that they should make sacrifices now to prevent that in the future.” (participant 1, FG Gaucher 1)

Fabry patients were willing to participate in trials if it would help future generations, even if they would not benefit themselves.

“I would hope for some freedom. Not even for myself, but for my son who is celebrating his birthday today. I would hope for some freedom for him.” (participant 1, FG Fabry 6)

Furthermore, the MPS III parents expressed their gratitude for patients and their parents who contributed to the progression of research by participating in previous clinical trials, even under uncertain circumstances in early stages.

“Just the fact that they are working on a solution, I am really grateful for that. […] If [gene therapy] works even partially, our child would benefit, and all the children [i.e. MPS III patients] who will come after might benefit. There have been a lot of children before our child who did the same [i.e. participating in trials]. Because of that we are where we are now regarding research and I am really grateful for that.” (participant 1, FG MPS III 2)

For adult MPS III patients their current situation regarding health and the opinion of their family and doctor played an important role in the decision whether to participate in gene therapy trials.

### Ethical aspects

Ethical aspects were mainly addressed at the end of the discussions. Different topics were discussed, such as religious beliefs and the boundaries to which gene manipulation should be restricted. Regarding costs, patients estimated gene therapy to be extremely expensive, which would be justified according to Gaucher and Fabry patients if it would be a one-time treatment with long term effectiveness since in that case it would possibly be less costly than long term ERT.

“What am I costing now, year after year? I assume that I would only be treated [with gene therapy] if it is really necessary. […] I think that would actually be cheaper.” (participant 2, FG Fabry 4)

MPS III patients and parents did not consider the costs and thought that it was up to the government and the pharmaceutical companies to negotiate.

### Ranking

**Fig. 2:**
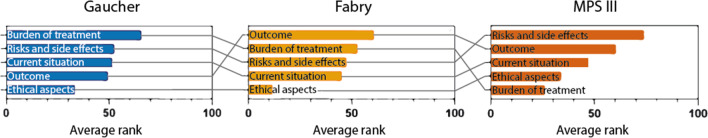
**Ranking of themes by Gaucher patients, Fabry patients and MPS III patients and parents.** Participants were asked to rank the factors that arose in their discussion group or interview from most influencing to least influencing the decision whether to undergo gene therapy. These factors were assigned to one of the five themes. A high ranked theme means participants attributed a lot of influence to the factors making up that theme

The ranking of the five themes is shown in Fig. 2. The greatest differences are found in the importance assigned to the *outcome* and *burden of treatment*. Gaucher and Fabry patients deemed the *burden of treatment* important, whereas MPS III patients and parents ranked this theme lower than *ethical aspects,* the theme ranked lowest overall. Fabry and MPS III patients and parents ranked *outcome* high, suggesting hope for improvement. Gaucher patients ranked *burden of treatment*, *risks and side effects* and *current situation* higher than *outcome*, which could indicate a more cautious attitude towards gene therapy. The rankings supported our findings from the focus group discussions and interviews.

## Discussion

In this study, focus group discussions and interviews were conducted to gain insight in the factors influencing the decision to undergo gene therapy in Gaucher type 1 patients, Fabry patients,  MPS III patients with attenuated disease and parents of (young) MPS III patients with or potentially developing severe manifestations. Participants’ views ranged from hesitancy to eagerness to choose for gene therapy, which seemed to be mostly related to disease severity and currently available treatment options. Patients with Gaucher disease, the disorder with the best treatment outcomes, were the most reluctant and expected at least similar effectiveness of gene therapy compared to ERT. For them, the biggest advantage would be not to be dependent on infusions every other week. Patients with Fabry disease were willing to accept more risks and uncertainty regarding effectiveness of gene therapy, hoping it will be more effective than their current therapeutic options. Parents of children with severe MPS III phenotypes, the condition with the worst prognosis and without current treatment options, considered every therapeutic option worthy to try, as this represented their only hope for improvement. Mildly affected adult MPS III patients hoped for stabilization of disease progression and a longer life expectancy, but were more cautious compared to parents of MPS III patients, because they valued their current health situation. Ranking of the factors that arose in the focus group discussions or interviews supported the presented conclusions.

To the best of our knowledge, this is the first study exploring patients’ needs and expectations regarding gene therapy in LSDs. Similar studies have been published for hemophilia [24, 25] in which it was shown that severely affected hemophilia patients had a more positive attitude towards gene therapy compared with less severely affected patients, which is in line with our findings. Moreover, a study reporting the experiences of hemophilia patients treated with gene therapy in several trials in the UK was recently published [26]. The authors conclude that patients need to understand the processes and implications of gene therapy prior to the treatment, since patients had experienced the side effects of immunosuppressive therapy as a troubling factor [26, 27].

In the discussions with parents of children with MPS III it was mentioned that the possibility of life extension as a result of gene therapy raises a dilemma. A parent wanted to be able to carry the responsibility for the child and was reluctant to put any burden on siblings, other family members or society. This is in line with previous findings of Shapiro et al. who studied the caregiver burden of parents with a child with MPS III and described a higher burden as children got older [28]. Another interesting finding was that several Fabry patients expressed the hope that gene therapy would have a positive effect on complaints that ERT cannot solve, such as fatigue and pain. Both examples underline the importance to involve patients in the development of new treatment modalities, as was previously concluded [29].

A limitation of this study was the online character of the focus group discussions due to the COVID-19 pandemic. This might have influenced the course of the discussion and non-verbal communication may have been missed, although during the evaluation of the focus group discussions participants said they had been able to express themselves properly. A second factor influencing the discussion was the uncertainty about several aspects of gene therapy (e.g. the precise procedure, risks and side effects). Despite this uncertainty, participants were able to elaborate on the different topics and explain which factors influence their decision whether to choose for gene therapy. An advantage was that it helped to have an open discussion in which the participants could freely express their thoughts.

Another limitation was the small sample size, and potential bias because of selection of participants who were interested in the subject and reflected perhaps a stronger opinion compared to non-interviewees. However, for rare diseases, a sample size of 39 patients and parents of patients with a LSD is relatively large. A strength of this study is the ranking of factors discussed during the focus groups or interviews. This was done individually by the participants after the conversations and confirmed our conclusions.

In conclusion, this study provides insights in patients’ needs and expectations regarding gene therapy in three different LSDs. It illustrates the importance of involving patients or their representatives in early stages of treatment development and clinical trial design in order to ensure that developed treatments and outcomes meet patients’ needs. The hesitant attitude of (most) Gaucher patients and some Fabry patients (mainly the ones that have no or limited disease manifestations) underlines the importance of exploring patients’ needs and expectations before using limited resources for the development of therapies for patient groups of which a significant subset may not be willing to undergo that specific therapy.

## Supplementary Information

**Additional file 1**. Topic list focus group discussions.

**Additional file 2**. Topic list interviews.

## Data Availability

The datasets used and/or analyzed during the current study are available from the corresponding author on reasonable request.

## Abbreviations

COREQ: Consolidated criteria for reporting qualitative research
ERT: Enzyme replacement therapy
FG: Focus group
HSCT: Hematopoietic stem cell transplantation
LSD: Lysosomal storage disease
MPS III: Mucopolysaccharidosis type III
SRT: Substrate reduction therapy
