# Supplementary material for: Patients’ view on gene therapy development for lysosomal storage disorders: a qualitative study
Source: Orphanet J Rare Dis. 2022 Oct 21;17:383. doi: 10.1186/s13023-022-02543-y (PMC9587648; doi:10.1186/s13023-022-02543-y)
Supplement: Supplementary file 1 — Additional file 1. Topic list focus group discussions. [file 13023_2022_2543_MOESM1_ESM.docx]

**Topic list focus group discussions**

Aim: to explore patients’ expectations and preferences regarding gene therapy for Gaucher disease, Fabry disease and Mucopolysaccharidosis type III using semistructured focus group discussions.

Introduction

- Welcome
- Introduction of investigators and participants
- Rules during discussion

Background information (part 1)

- What are the first associations that come to mind regarding gene therapy?
- What knowledge do the participants have regarding gene therapy options for their specific lysosomal storage disorder (LSD)?
- What knowledge do the participants have regarding the treatment options for their specific LSD?

*At the end of part 1, the information provided to all participants in advance can be discussed with a doctor specialized in inborn errors of metabolism, as well as questions that arose during the first part of the discussion in order to ensure that all participants are able to contribute to the discussion in part 2.*

Patients’ needs and preferences regarding gene therapy (part 2)

- What should be the goal of gene therapy?
- Which factors play a role in the decision to undergo gene therapy?

First an open question is asked, then the factors listed below are discussed:

- - Comparison to current treatment options
  - Potential complications and side effects
  - Burden of treatment (mode of administration, additional treatments, hospital visits, regimen)
  - Efficacy (potentially curative, potentially effective, duration of efficacy)
  - Costs and ethical considerations
- Would the participants undergo gene therapy if it would be available at this moment?
  - Factors that would contribute to that decision

*At the end of part 2, the assistant moderator summarizes the factors mentioned during the discussion. These factors will be sent to the participants by email and they are asked to rank them based on importance.*

Closing remarks
