# Supplementary material for: Patients’ view on gene therapy development for lysosomal storage disorders: a qualitative study
Source: Orphanet J Rare Dis. 2022 Oct 21;17:383. doi: 10.1186/s13023-022-02543-y (PMC9587648; doi:10.1186/s13023-022-02543-y)
Supplement: Supplementary file 2 — Additional file 2. Topic list interviews. [file 13023_2022_2543_MOESM2_ESM.docx]

**Topic list interviews**

Aim: to explore patients’ expectations and preferences regarding gene therapy for Gaucher disease, Fabry disease and Mucopolysaccharidosis type III using semistructured interviews.

Introduction (part 1):

- Welcome
- Introduction of investigators and participant
- Rules during discussion

Patients’ needs and preferences regarding gene therapy (part 2)

- Which factors play a role in the decision to undergo gene therapy?
  - Positive factors with respect to the current situation?
  - Negative factors with respect to the current situation?
- How does the participant feel about the burden of the different types of treatment? Where would he/she draw the line?
- How does the participant feel about the possibility of (temporal) side effects?
- How does the participant feel about the possibility that gene therapy has no or limited effect?
- If gene therapy would be available to the participant at this moment, would he/she wish to undergo it? Which factors would contribute to that decision?

*At the end of part 2, the assistant moderator summarizes the factors mentioned during the discussion. These factors will be sent to the participant by email and he/she is asked to rank them based on importance.*

Closing remarks
